# Risk assessment of Ebola Reston virus in humans in the Philippines

**DOI:** 10.5365/wpsar.2017.3.004

**Published:** 2019-07-05

**Authors:** Johnette A Peñas, Mary Elizabeth Miranda, Vikki Carr de los Reyes, Ma. Nemia L Sucaldito, Rio L Magpantay

**Affiliations:** a Department of Health, Philippines.

## Abstract

**Objective:**

There have been five documented outbreaks of Ebola Reston virus (RESTV) in animals epidemiologically linked to the Philippines. This assessment was conducted to determine the risk of RESTV occurring in humans in the Philippines and its potential pathogenicity in humans.

**Methods:**

The World Health Organization *Rapid Risk Assessment of Acute Public Health Events Manua*l was used for the assessment. A literature review was done and a risk assessment matrix was used for the risk characterization of the outbreaks in the Philippines. The risk assessment was conducted by the Philippines Field Epidemiology Training Program.

**Results:**

The risk of RESTV occurring in humans in the Philippines and its potential pathogenicity in humans were both assessed as moderate. Animals involved in RESTV outbreaks in the Philippines were non-human primates and domestic pigs. The presence of RESTV in pigs poses a possibility of genetic evolution of the virus. Although RESTV has been identified in humans, there was no death or illness attributed to the infection. The Philippines Inter-agency Committee on Zoonoses oversees collaboration between the animal and human health sectors for the prevention and control of zoonoses. However, there is no surveillance of risk animals or previously affected farms to monitor and facilitate early identification of cases.

**Discussion:**

The moderate risk of RESTV recurring among humans in the Philippines and its potential pathogenicity in humans reinforces the need for early detection, surveillance and continued studies of RESTV pathogenesis and its health consequences. The One Health approach, with the involvement and coordination of public health, veterinary services and the community, is essential in the detection, control and management of zoonosis.

Ebola Reston virus (RESTV) is one of the six virus species of the Ebola virus in the family Filoviridae. (*1*, *2*) Although three filoviruses have been identified in animals in Asia, (*3*, *4*) RESTV is the only filovirus isolated from Asia that is known to infect humans. (*5*) There have been five documented RESTV outbreaks in animals epidemiologically linked to the Philippines. (*6*, *7*) RESTV was detected in non-human primates (NHPs) in the periods 1989–1990, 1992–1993 and 1996; (*6*, *8*) in pigs in 2008–2009; (*6*, *9*, *10*) and again in NHPs in 2015. (*7*) These NHPs were cynomolgus macaques used for preclinical research, drug development, disease modelling, experimental infections, and biological production, with breeders being collected from wildlife trapping areas mostly in southern Philippines. (*6*) Four of of the five outbreaks were investigated by the Philippines Field Epidemiology Training Program (FETP). (*7*-*12*)

There is concern in the Philippines that RESTV will continue to occur in animals with spillover into humans and could one day become pathogenic to humans. (*1*, *10*, *12*, *13*) It has been hypothesized that ongoing, undetected RESTV infections and replication in pigs and other animals could result in the emergence of more pathogenic viruses in humans and/or livestock. (*13*) Therefore, a risk assessment was conducted to determine the risk of further occurrence and potential pathogenicity of RESTV in humans in the Philippines.

## Methods

The World Health Organization (WHO) *Rapid Risk Assessment of Acute Public Health Events Manual* (*14*) was used for this risk assessment. It included conducting hazard, exposure and context assessments to determine the level of risk. The WHO risk assessment matrix was used to characterize the level of risk based on the combined estimate of likelihood and consequences of the event.

The risk assessment was conducted by the Philippines FETP. The team comprised public health specialists in applied epidemiology with expertise in epidemiology, infectious diseases, risk communication and emergency planning. Several team members were part of the response teams for the RESTV investigations.

To enable an evidence-based risk assessment, literature reviews were conducted on articles with RESTV data. The archives of the Epidemiology Bureau library in the Department of Health (DOH) were searched for all RESTV-related investigations conducted by FETP fellows from 1989 to 2015. A MEDLINE search using PubMed was conducted using the search terms “Ebola” and “Reston” with search dates between 1 November 1989 and 1 November 2016. Only articles containing data on RESTV studies and its occurrence were reviewed. Information on the pathogenicity of RESTV was published in a 2009 WHO meeting report on Ebola Reston pathogenicity in humans. (*15*) This informal meeting was conducted to provide guidance on responding to queries related to RESTV pathogenicity in humans.

## Results

### Literature review

Seven RESTV-related investigation reports by FETP fellows were identified. (*7*-*12*, *16*) A MEDLINE search produced 129 scientific and medical abstracts and full-text reports, and 19 were relevant to the risk assessment. (*1*-*6*, *13*, *17*-*31*) These 26 reports are all full-text reports; 12 studies were conducted following the five RESTV outbreaks in animals epidemiologically linked to the Philippines and one occurring in China. The remaining 14 reports were serological/molecular studies in humans, monkeys and/or bats (9); genome virus analyses (2); ecologic niche modelling of outbreaks (1); study on filovirus survival ability (1); and a review of RESTV in the Philippines (1).

### Hazard assessment

#### RESTV outbreaks in animals epidemiologically linked to the Philippines

There were five documented RESTV outbreaks in animals epidemiologically linked to the Philippines. (*6*, *7*) The first outbreak was in November 1989 in Reston, Virginia, United States of America (USA) when quarantined NHPs from the Philippines became ill and died. (*12*, *22*) Epidemiological investigation in the monkey-breeding facilities in the Philippines at that time revealed RESTV-infected animals in the facilities. (*11*, *12*) This was the first-ever Ebola virus detected outside of Africa and was the first known natural infection of Ebola virus in NHPs. (*6*)

From 1992 to 1993, RESTV was detected in an NHP quarantine facility in Sienna, Italy, and infected NHPs were again traced back to the Philippines. (*6*) In March 1996, imported macaques from the Philippines tested positive for RESTV in another facility in Texas, USA. (*8*) In October 2008, RESTV RNA was unexpectedly identified in pig tissue samples sent from the Philippines for porcine reproductive and respiratory syndrome (PRRS) strain analysis in the Plum Island Animal Disease Center in Greenport, New York, USA. (*9*) Joint investigation by the FETP, the Bureau of Animal Industry and international experts revealed that the positive samples came from two commercial pig farms. (*10*) RESTV was detected in the Philippines in September 2015 in monkeys bound for export. (*7*) Risk communication was done to allay public fears. It was emphasized that RESTV is the mildest type of Ebola and does not pose the same threat as the Democratic Republic of the Congo Ebola virus in West Africa. (*32*)

#### Serological testing in human and animals

Across these five RESTV outbreaks in animals, a total of 1445 humans were tested for RESTV; all had been occupationally exposed to NHPs or pigs or were close contacts of seropositive persons. (*6*-*11*) A total of 105 people (7%) were positive; most (100/105, 95%) were pig handlers and abattoir workers from the 2008–2009 investigations after the detection of RESTV in pigs in Pangasinan and Bulacan. (*9*, *10*)

The highest number of animals testing positive for RESTV was in the 1989–1990 investigation when 142/179 (79%) NHPs tested in the Philippines were antibody positive and 141/279 (51%) were antigen positive (**Table 1**). (*6*-*12*) Two per cent (3/186) of occupationally exposed animal handlers tested positive. (*15*) This serosurvey was initiated following the report of RESTV-infected macaques in the USA from two major export facilities in the Philippines. (*11*, *12*)

**Table 1:** Ebola Reston virus laboratory results and signs and symptoms, 1989 to 2015

Year of outbreak	NHP			Pig			Human		
	Antibody	Antigen/ RNA	Signs and symptoms	Antibody *n* = 153	Antigen/ RNA	Signs and symptoms	Antibody	Signs and symptoms	Population group
1989–1990	142 (79%) ^a^ *^(^**9**^)^*	141 (51%) ^b^ *^(^**9**^)^*	Diarrhoea, respiratory symptoms, wounds, bleeding, weakness, loss of appetite *^(^**9**^)^*	-	-	-	3 (3%) ^c^ *^(^**8**^)^*	No illness *^(^**8**^)^*	Animal handler, veterinarian *^(^**8**^)^*
1992–1993	-	-	-	-	-	-	2 (2%) ^d^ *^(^**3**^)^*	No Illness *^(^**3**^)^*	Breeding and export facility employees *^(^**3**^)^*
1996	3 (2%) ^d^ *^(^**5*, *25**^)^*	131 (47%) ^e^ *^(^**5*, *25**^)^* 6 (2%) ^f^ *^(^**23**^)^*	Signs of gastrointestinal infection, anorexia, paralysis *^(^**23**^)^*	-	-	-	1* (1%) ^d^ *^(^**5**^)^*	No illness *^(^**5**^)^*	Breeding and export facility employee *^(^**5**^)^*
2008–2009	-	-	-	153 ^d^ *^(^**7**^)^*	28 ^f^ *^(^**6**^)^*	Clinical signs resembling PRRSV *^(^**7**^)^*	100 (95%) ^d^ *^(^**3*, *6*, *7**^)^*	No illness *^(^**6*, *7**^)^*	Abattoir workers and pig handlers *^(^**6*, *7**^)^*
2015	34 (19%) ^g^ *^(^**4**^)^*	1 (0%) ^f^ *^(^**4**^)^*	Anorexia, dehydration, petechial haemorrhage *^(^**4**^)^*	-	-	-	0 (0%) ^g^ *^(^**4**^)^*	-	Workers at monkey-holding facilities *^(^**4**^)^*
Total	179	279	-	153	28	-	105	-	-

NHP: non-human primate; PRRSV: porcine reproductive and respiratory syndrome virus.

a Indirect fluorescent antibody assay cut-off of ≥ 1:16.

b Antigen detection by enzyme-linked immunosorbent assay (ELISA).

c Indirect fluorescent antibody test cut-off of ≥ 1:256.

d IgG antibody by ELISA.

e Antigen detection test by enzyme immunosorbent assay (EIA) cut-off of ≥ 1:16.

f Reverse-transcriptase polymerase chain reaction (RT–PCR).

g IgG and IgM antibody by ELISA.

* Same person already IgG positive in 1992–1993.

Note: Not all RESTV-positive non-human primates and pigs are symptomatic.

#### Clinical factors

Signs and symptoms manifested by RESTV-positive NHPs were diarrhoea, respiratory symptoms, wounds, bleeding, weakness, gastrointestinal infection, anorexia and paralysis. (*6*, *12*, *26*) Some of the RESTV-positive pigs had clinical signs that resembled PRRS virus infections. (*1*, *9*, *10*, *13*) However, it was also observed that RESTV can be asymptomatic in NHPs and pigs (**Table 1**). (*7*, *8*, *18*, *26*, *27*) Some animals infected with RESTV were shown to be immunocompromised or having a coinfection. (*1*, *24*) These coinfections included simian haemorrhagic fever (NHPs, 1989–1990), (*6*) PRRS (pigs, 2008–2009) (*9*, *10*, *24*) and measles (NHPs, 2015). (*7*)

In humans, there have been no deaths or illness attributed to RESTV infection; rather, infection results in a very mild illness. (*6*, *8*-*11*) Therefore, RESTV does not pose the same public health threat as the African Ebola virus subtypes. (*11*, *27*) As the evidence available relates only to healthy adults, further studies are needed to clarify whether these health effects would be the same for all population groups, such as those with underlying medical conditions, immunocompromised individuals, pregnant women and children. (*15*) However, these population groups may be less likely to be in contact with infected NHPs, pigs and bats compared to the other groups (healthy, no special condition) as they probably spend more time indoors and are less likely to engage in activities exposing them to the said animals. RESTV in domestic pigs also increases the opportunity of pig-to-human interspecies transmission because of their frequent and close level of contact. (*15*)

According to the WHO experts consultation on RESTV pathogenicity in humans, the virus is genetically diverse, (*15*) and slight changes in its genetic sequence could result in a more virulent virus in humans. (*21*) When there was interspecies transmission (e.g. monkeys to pigs), RESTV was thought to evolve more rapidly. (*1*, *15*) In the affected farm in the 2008–2009 RESTV outbreak in pigs, there was a 0.079% genetic diversity of the virus over a one-year period, and simultaneous samplings in another farm in 2008 found the divergence to be about 4.5%. (*18*) The presence of RESTV in pigs poses a high potential for genetic evolution since domestic pigs, as compared to NHPs and bats, have frequent contact with humans. (*9*, *10*, *15*) With no surveillance for RESTV in pigs, bats and NHPs in the wild, it is possible that there is undetected ongoing circulation of the virus in animals, providing opportunity for continued genetic evolution with passage, adaptation and its possible natural selection. (*13*, *15*) However, there is no research on RESTV virulence factors, and it is difficult to tell based on genetic sequence data which RESTV strain might be pathogenic in humans. (*15*)

### Exposure assessment

#### Geographic distribution

Animal and human infections of RESTV have occurred in five provinces and two cities in the Philippines. (*7*, *8*, *10*, *11*) Laguna province has had cases in humans and NHPs. (*6*, *8*, *11*) The provinces of Pangasinan and Bulacan have had cases in humans and pigs. (*9*, *10*) Nueva Ecija province and Valenzuela City have had cases only in humans, (*9*, *10*) and Batangas province and Parañaque City have had cases only in NHPs. (*7*-*12*) Some of the infected NHPs in Laguna were caught in the wild on the island of Mindanao. (*8*) While the geographic origin of RESTV is hypothesized to be South-East Asia and the Philippines, (*22*) distribution has been shown to be widespread as it also occurs outside Asia. (*5*, *23*)

Filoviruses have been identified in Africa, Europe and Asia. (*22*, *23*, *27*, *30*, *33*, *34*) Serological studies in other countries from 1990 to 2011 found RESTV in humans in Germany, (*23*) pigs and bats in China, (*19*, *24*) orangutans in Indonesia (*20*) and bats in Bangladesh (*25*) and South Africa. (*5*)

#### Modes of transmission

Humans who tested positive in serological studies had daily exposure to pigs or NHPs. (*6*-*11*) The mode of transmission of RESTV to humans is most likely through close or direct contact with infected animals’ secretions, blood, organs or bodily fluids. (*9*, *10*, *15*) It is uncertain whether RESTV can be transmitted to humans through inhalation of infected respiratory secretions, but it has been described in NHPs. Some studies also found that experimentally infected pigs with RESTV can shed virus from the nasopharynx, suggesting a route of transmission by aerosol or droplet contact. Further investigations are needed for clarification. (*13*, *15*, *18*, *21*, *27*, *31*)

There is no indication of human-to-human transmission of RESTV. In the 1989–1990, 1996 and 2008–2009 investigations, several contacts of RESTV-positive individuals all tested negative. (*6*) However, human-to-human transmission is potentially possible if an individual were to become viraemic and symptomatic. This has occurred in other filoviruses, and there was a documented three-day viraemia in a human with RESTV infection. (*15*)

#### Natural reservoirs of RESTV

Bats have been identified as natural reservoirs of filoviruses, including Ebola and Marburg viruses. (*5*, *13*, *17*, *19*, *25*, *29*, *30*) In the Philippines, there is evidence of RESTV infections in bats in Quezon City and in the provinces of Bataan, Bulacan and Quezon. (*29*, *30*) It is possible that RESTV was transmitted to NHPs and pigs from bats since bats inhabit many areas of the country, including the regions around the affected facilities in Bulacan, Pangasinan and Batangas. (*7*, *9*, *10*, *17*, *30*) In a 2010 risk assessment of bat exposure among people in Orani, Bataan, bat meat consumption (93%), presence of bats near house (90%) and handling of bats (77%) were common. (*16*)

### Context assessment

#### Policy factors

The capacity of the Philippines to detect and respond to the RESTV outbreak is limited but has improved over time. The Philippine National Reference Laboratory for Emerging and Re-emerging Infectious Diseases (NRL-EREID) has the ability to test both human and animal samples for RESTV. Testing for RESTV in humans is done when outbreaks occur in animals. Currently, there are two monkey-holding facilities in the Philippines, and only NHPs for export are tested for filovirus. The Bureau of Animal Industry and the World Organization for Animal Health do not consider RESTV to be a priority or notifiable animal disease. (*35*, *36*)

#### Environmental factors

In the late 1970s there was a marked increase in human population along with logging and deforestation in the Philippines. (*18*) Deforestation and other landscape transformations result in more direct and indirect human contact with primates and bats and alter geographic distribution of animals, leading to increased risk of old and new zoonosis. (*37*)

The total swine population in the Philippines has reached 13.13 million. (*38*) The pig industry can expose pig handlers and abattoir workers to viruses. In the 2008–2009 RESTV outbreak, the majority of RESTV-positive pig handlers wore only rubber boots as personal protective equipment. (*9*, *10*) Despite the 2008–2009 outbreak in pigs, no surveillance has been conducted in the affected farms to determine if transmission stopped after pig depopulation. (*10*) The risk of contaminated meat entering the food-chain is possible. This is a potential route of transmission with an urgent need for risk assessment. (*15*)

#### Technical and scientific factors

The first three RESTV outbreaks were initially detected in NHP quarantine facilities in Virginia (USA), Italy and Texas (USA) (*6*, *8*, *11*, *12*) and were subsequently traced back to one facility in the Philippines. (*1*, *17*) In 2008–2009, RESTV was coincidentally detected in pig tissues sent to the USA for PRRS strain analysis. (*9*, *10*) In 2015, RESTV was identified in NHPs bound for export; a filovirus test conducted during the 31-day quarantine yielded positive results in nine apparently healthy NHPs. (*7*) This prevented the exportation of yet another infected NHP from the Philippines. Of the five RESTV outbreaks, only one was detected in the Philippines by the DOH-EREID. Testing of NHPs for export is not sufficient to identify all cases in animals. Animal surveillance and laboratory testing are necessary to capture RESTV cases in animals.

Since previous human cases of RESTV were asymptomatic, possible cases may seek medical care or testing only if another outbreak occurred in animals. Thus, the likelihood that cases are identified is low. The Philippines One Health (*33*) concept recognizes the need for intersectoral collaboration in public health, social sciences, medicine, veterinary sciences and agriculture to mitigate complex socioecological drivers that contribute to ill health. (*37*) The Philippines One Health approach addressed RESTV outbreaks in NHPs and pigs; various agencies took part in virus detection, NHP and pig depopulation and bat surveillance. In 2011, The Philippines Inter-agency Committee on Zoonoses was created to establish animal and human health sector collaboration for the prevention and control of zoonoses. (*33*) The NRL-EREID programme also highlights strategic priorities to prevent and control diseases from becoming public health problems. Priorities include resource management, coordinated networks of facilities and managing information to enhance disease surveillance. (*34*)

#### Economic factors

RESTV outbreaks resulted in NHP depopulation and closing of two monkey-holding facilities in 1996 and 2015. (*7*, *26*, *28*) Also, 6210 pigs were culled in 2008 to prevent the spread of the virus to other pigs and to reduce exposure to abattoir workers. (*10*) These control measures during RESTV outbreaks greatly affected livelihoods and the economy.

### Risk characterization

Using the information from the risk assessment, the risk of RESTV occurring in humans in the Philippines was considered moderate, based on that it is likely to occur; however, the consequences would be minor (**Table 2**). The risk of potential pathogenicity in humans was also assessed as moderate. While the consequences of RESTV human pathogenicity could be major if it became highly pathogenic, the very low likelihood makes it unrealistic to consider the resultant overall risk as high as formally dictated by the risk assessment tool. Accepting that the risk assessment tool allows for a certain degree of judgment and flexibility, we thus consider the overall risk as moderate (**Table 3**).

**Table 2:** Risk analysis matrix for the assessment of risk for RESTV further occurrence

Likelihood	Consequences				
	Minimal	Minor	Moderate	Major	Severe
**Almost certain**	**Low**	**Moderate**	**High**	**Very high**	**Very high**
**Highly likely**	**Low**	**Moderate**	**High**	**Very high**	**Very high**
**Likely**	**Low**	**MODERATE**	**High**	**High**	**Very high**
**Unlikely**	**Low**	**Low**	**Moderate**	**High**	**High**
**Very unlikely**	**Low**	**Low**	**Moderate**	**High**	**High**

**Table 3:** Risk analysis matrix for the assessment of risk for RESTV pathogenicity

Likelihood	Consequences				
	Minimal	Minor	Moderate	Major	Severe
**Almost certain**	**Low**	**Moderate**	**High**	**Very high**	**Very high**
**Highly likely**	**Low**	**Moderate**	**High**	**Very high**	**Very high**
**Likely**	**Low**	**Moderate**	**High**	**High**	**Very high**
**Unlikely**	**Low**	**Low**	**Moderate**	**High**	**High**
**Very unlikely**	**Low**	**Low**	**Moderate**	**High**	**High**

Note: Risk analysis matrix was adapted from WHO **^(11)^**.

The level of confidence on this risk assessment is low to medium based on the data presented. Information on the hazard, exposure and context assessments was based on different sources, which include first-hand epidemiological investigation reports and peer-reviewed articles; however, there was little information on surveillance, epidemiological and clinical data. These limitations could alter the understanding of RESTV and the risks to humans.

## Discussion

In this risk assessment, the risk of RESTV occurring in humans in the Philippines and its potential pathogenicity in humans were both assessed as moderate.

RESTV in humans was deemed likely to occur since RESTV infection has been detected in humans, pigs, bats and NHPs from different locations within the Philippines. With pigs as a host of RESTV, the likelihood of further human contact with infected animals is high, and the likelihood of the virus entering the food-chain is possible. If RESTV remains non-pathogenic to humans, the consequence will remain minor.

To date, there has been no evidence of RESTV pathogenicity in humans and no deaths or illness among the 105 RESTV-positive humans. (*6*-*12*) However, some changes in the genetic sequence of RESTV could result in a virus more virulent in humans, especially if there is interspecies transmission. (*15*) Should RESTV spread and become pathogenic in humans in the Philippines, the health consequences would escalate. Further evaluation would be needed if it occurred to establish the evolving risks. Response to the event would depend on the RESTV pathogenicity.

Enhanced surveillance is needed, and exposure of humans to animals and environmental sources should be controlled. Strict implementation of quarantine and filovirus testing of all NHPs for export should be continued. Sentinel testing of other NHPs within the Philippines should be considered to detect potentially latent diseases and prevent their introduction into a larger laboratory animal colony. Serological testing of domestic pigs in areas with a history of RESTV should be considered, especially if there are unusual pig deaths. Testing would allow detection of the virus before it enters the food-chain, thus limiting the possible emergence of a more pathogenic strain due to replication in livestock.

There are limitations to our risk assessment. Current risk assessment is based on post-hoc reports on incidental findings of the virus through exported animals or tissue samples; therefore, the true epidemiological and clinical profile of infections in animals or humans are unknown. There has been no surveillance or serological surveys of domestic pigs, NHPs or people occupationally exposed to determine if the transmission has stopped or if there is ongoing circulation of the virus. With no ongoing surveillance data, current/baseline infection rates and/or viral genetic evolution are not established. As a result, it is impossible to know the true prevalence of the infection or be alerted for further outbreaks. We used information from the WHO consultation on Ebola Reston pathogenicity in humans, which was conducted after RESTV was detected in pigs (2009), and there have been further incidents since then. Aside from FETP scientific papers, literature review was limited to MEDLINE. Finally, risk assessments by their nature are somewhat subjective; therefore, other risk assessment approaches may have different outcomes.

Future studies will shed light on RESTV pathogenicity and its consequences on animal and human health. Follow-up and serological studies on RESTV-positive humans should be done. Research studies on RESTV epidemiology, viral genetics, reservoir, potential hosts, clinical disease in humans and animals including incubation period, risk factors for infection, pathogenesis in coinfection and immunocompromised hosts, mechanism and prevention of transmission and public health impact should also be undertaken.

Our assessment showed that the risk of RESTV occurring again in humans in the Philippines is moderate, and the risk of potential pathogenicity in humans is also moderate. The Philippines must not be complacent about the detection of RESTV. Applying and intensifying the One Health approach by doing surveillance, research, risk communication, risk reduction measures, and collaboration involving at-risk communities and human and animal health sectors should be initiated and continued for preparedness and response for potential RESTV outbreaks.
